# Comparison of Inpatient and Outpatient Cardiac Rehabilitation Following Myocardial Infarction

**DOI:** 10.3390/jcm14093007

**Published:** 2025-04-26

**Authors:** Piotr Jankowski, Roman Topór-Mądry, Paweł Kozieł, Daniel Cieśla, Urszula Cegłowska, Monika Burzyńska, Zbigniew Eysymontt, Radosław Sierpiński, Jarosław Pinkas, Mariusz Gąsior

**Affiliations:** 1 Department of Internal Medicine and Geriatric Cardiology, Centre of Postgraduate Medical Education, 02-507 Warsaw, Polandtragez88@wp.pl (P.K.); 2 Department of Epidemiology and Health Promotion, Centre of Postgraduate, School of Public Health, 01-826 Warsaw, Poland; uceglowska@gmail.com; 3 Department of Epidemiology and Population Studies, Institute of Public Health, Jagiellonian University Medical College, 31-008 Kraków, Poland; roman.topor-madry@uj.edu.pl; 4 3rd Department of Cardiology, Faculty of Medical Sciences in Zabrze, Medical University of Silesia, 40-055 Katowice, Poland; d.ciesla@sccs.pl (D.C.); m.gasior@op.pl (M.G.); 5 Department of Epidemiology and Biostatistics, Division of Social and Preventive Medicine, Medical University of Lodz, 90-151 Lodz, Poland; 6 Silesian Centre of Cardiac Rehabilitation and Prevention, 43-450 Ustron, Poland; gable.zibi@gmail.com; 7 National Centre for Health Policy and Research on Health Inequalities—Cardinal Stefan Wyszynski University in Warsaw, 01-938 Warsaw, Poland; r.sierpinski@uksw.edu.pl; 8 Centre of Postgraduate Medical Education, School of Public Health, 01-826 Warsaw, Poland; jaroslaw.pinkas@cmkp.edu.pl

**Keywords:** cardiac rehabilitation, coronary artery disease, myocardial infarction, secondary prevention

## Abstract

**Background:** Models of second-phase cardiac rehabilitation (CR) following myocardial infarction (MI) differ across countries. The aim of this study was to compare outcomes in MI survivors participating in outpatient and inpatient CR programs. **Methods:** All patients hospitalized for acute MI in Poland between October 2017 and December 2018 (n = 96,634) were included in the study. Among them, 4411 patients were referred to and commenced outpatient CR, whereas 11,626 patients started inpatient CR within 30 days following discharge. **Results:** The mean follow-up period was 332.8 ± 128.1 days. Younger age, male sex, and a history of cancer were associated with a higher probability of participating in outpatient CR, whereas diabetes, heart failure, chronic kidney disease, chronic obstructive pulmonary disease, ST-elevation MI, and myocardial revascularization were associated with a lower likelihood of outpatient CR participation. Participation in outpatient CR was linked to a reduced risk of all-cause mortality, in both univariable (hazard ratio [95% confidence interval]: 0.37 [0.26–0.51]) and multivariable analyses (0.53 [0.38–0.74]). Outpatient CR was also associated with a lower risk of death, MI, or stroke (0.57 [0.48–0.67] in univariable analysis and 0.72 [0.61–0.84] in multivariable analysis), as well as a lower risk of death or cardiovascular hospitalization (0.78 [0.73–0.84] and 0.85 [0.80–0.91], respectively). **Conclusions:** Outpatient CR following MI tends to occur alongside a better prognosis compared to inpatient programs.

## 1. Introduction

Myocardial infarction (MI) continues to pose a significant challenge to healthcare systems. The high number of hospital admissions and the associated costs of treating acute MI patients place a considerable economic strain on medical resources. In Poland, more than 80,000 individuals experience acute MI annually, with one-year mortality rates exceeding 17% [1]. Additionally, nearly half of all MI patients require rehospitalization within a year for various medical reasons [2,3]. Advancements in medical technology, modern diagnostic methods, and overall improvements in public health, following Poland’s systemic transformation in 1989, have contributed to a substantial decline in mortality due to acute myocardial infarction (AMI) [4]. The standardized death rate for AMI decreased from 115.9 per 100,000 in 2000 to 33.0 in 2022. Despite these positive trends, further efforts are required to enhance the public awareness of early prevention strategies and to ensure comprehensive cardiac rehabilitation (CR) after MI, which could further reduce mortality rates. The latest European Society of Cardiology (ESC) and European Association for Cardio-Thoracic Surgery (EACTS) guidelines for post-revascularization cardiac care emphasize the importance of a multimodal management approach, which has been proven to enhance clinical outcomes and survival in AMI patients [5]. CR is strongly recommended and considered as crucial for invasive and non-invasive medical treatments [6]. CR is cost-effective, widely accessible, and associated with reduced cardiovascular morbidity and mortality [6,7,8,9]. Its benefits include improvements in physical activity, exercise capacity, psychological well-being, health-related quality of life, and risk factor control [10,11,12]. Current clinical practice guidelines recommend referral to CR following MI [10]. Although more than 80% of countries offer CR, the availability and structure of phase II CR vary worldwide [13,14,15,16]. In some countries, outpatient CR is the standard [17], whereas in others, it is primarily delivered as an inpatient (residential) program [11,16,18]. Additionally, some countries provide home-based or community-based CR programs [10,11]. While inpatient CR is often recommended for high-risk patients, in many countries, low-risk patients are still referred to residential CR [10,19,20]. The clinical benefits of CR following MI are well documented [6,8,9,10,11,20,21,22]. Although most published studies focus on outpatient CR, evidence suggests that inpatient CR is also associated with improved outcomes following MI [20,23,24,25]. However, no published analysis has directly compared outpatient and inpatient CR after MI. Both the European Society of Cardiology (ESC) guidelines and the American Heart Association (AHA) recommendations advocate for initiating cardiac rehabilitation (CR) as soon as possible after MI, preferably within the first 30 days. Early rehabilitation reduces both short-term and long-term mortality following MI [26]. The early introduction of exercise and therapy helps minimize left ventricular remodeling and improves cardiovascular performance [27]. Additionally, it lowers the risk of recurrent MI and cardiovascular-related rehospitalization. Patients who begin rehabilitation within 30 days have a significantly lower risk of rehospitalization due to cardiac causes. Early intervention also enhances medication adherence and encourages the adoption of a healthy lifestyle [28]. The SMARTCARE study demonstrated that patients who started CR within 14–30 days post-MI exhibited significantly better functional outcomes and lower mortality rates compared to those who initiated CR later or did not participate at all [29]. Based on this evidence and these recommendations, we analyzed the effects of CR when initiated within 30 days following discharge. Therefore, our study aimed to compare inpatient and outpatient cardiac rehabilitation following myocardial infarction among this group of patients.

## 2. Materials and Methods

### 2.1. Study Design and Population

This study included all adult patients hospitalized for acute MI in Poland between 1 October 2017 and 31 December 2018, who were referred to either inpatient or outpatient CR within 30 days following hospital discharge. Patients undergoing CR were classified into two distinct groups based on the setting in which they receive their rehabilitation program: inpatients and outpatients. Inpatients were defined as patients who remained hospitalized during the cardiac rehabilitation program. Outpatients participated in cardiac rehabilitation while living at home, attending structured CR sessions at a hospital, clinic, or rehabilitation facility several times per week. They did not require continuous hospitalization.

Exclusion criteria were as follows: patients aged < 18 years, patients who died during hospitalization, patients who initiated CR > 30 days after discharge, and patients who died or were rehospitalized for cardiovascular reasons within 30 days after discharge.

Medical histories were retrieved from the National Health Fund (NHF) database, which contains comprehensive records of hospitalizations, outpatient visits, and medical diagnoses. A diagnosis (e.g., hypertension, atrial fibrillation or other disease) was considered confirmed if it had been documented in the NHF system by any healthcare provider, including hospitals or outpatient clinics. CR participants were based on NHF records, which specify the type of rehabilitation program received and the setting in which it was conducted. Survival status was determined using national mortality records, while recurrent hospitalizations (including MI and stroke) were identified through the NHF database. A hospitalization was defined as an admission lasting >24 h, unless the patient died within 24 h.

Ethics committee approval was not needed as the authors analyzed the national databases. Informed consent was not required.

### 2.2. Endpoints

The primary endpoint was defined as death from any cause, whereas secondary endpoints were all-cause death or hospitalization due to any cardiovascular disease and all-cause death or myocardial infarction or stroke.

### 2.3. Statistical Analysis

Continuous variables are presented as the mean ± standard deviation, while categorical values are presented as percentages. The Shapiro–Wilk test was used to check the normality of the distribution. Normally distributed continuous variables were compared using the Student t test. The Mann–Whitney U test was used in the case of variables without a normal distribution. The Pearson χ2 test was applied to all categorical variables. A *p* value of less than 0.05 was considered statistically significant.

Propensity score matching using nearest neighbor matching (1:2) with replacement, based on the variables listed in Table 1, was applied to create comparable groups of patients participating in outpatient and inpatient CR programs. The propensity score was calculated using logistic regression. Balance between the matched groups was assessed by examining the standardized differences, with a difference of <10% considered acceptable. We used the Kaplan–Meier method to construct unadjusted survival curves for each endpoint and performed log-rank tests to evaluate differences between matched cohorts. The association between participation in outpatient CR and the risk of endpoints was assessed using Cox proportional hazard regression analysis. The proportional hazard (PH) assumption of the Cox regression model was evaluated using scaled Schoenfeld residuals. A stepwise analysis was conducted, starting with all variables presented in Table 1, with a probability value of >0.05 used for variable selection. Subgroup analysis was performed to examine the relationship between participation in outpatient CR and outcomes in the multivariable model. The test for interaction within the Cox model was used to compare hazard ratios between the analyzed subgroups. Additionally, a multivariable stepwise logistic regression analysis was conducted to identify independent factors associated with participation in outpatient CR. As part of the sensitivity analysis, we repeated the analyses after excluding all patients who experienced an endpoint within 90 days following discharge. All statistical analyses were performed using the STATISTICA 13 software (TIBCO Software, Palo Alto, CA, USA).

## 3. Results

**Figure 1:**
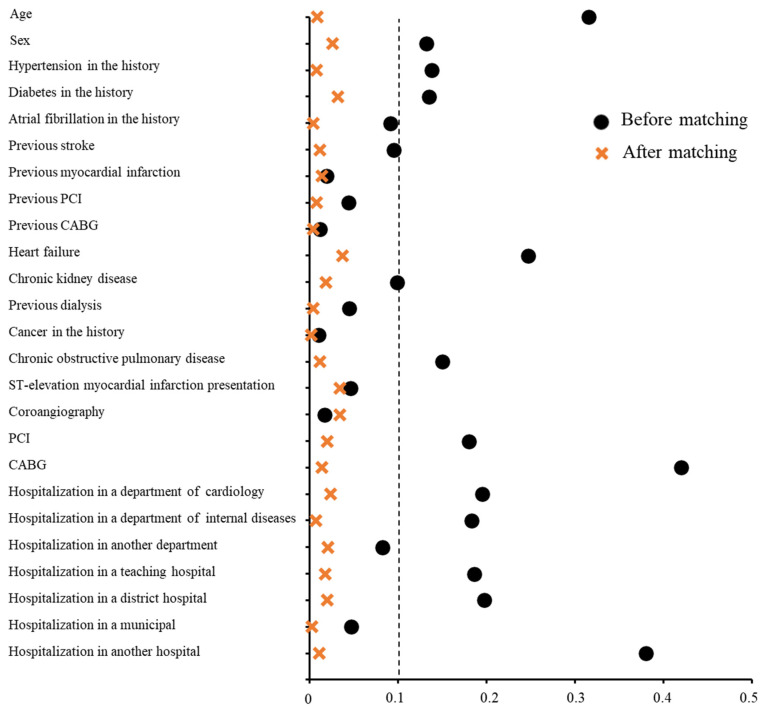
Standardized differences before and after propensity score matching, comparing covariate values for patients participating in inpatient and outpatient cardiac rehabilitation.

Overall, 4411 patients were referred to and initiated CR in an outpatient setting, while 11,626 patients were referred to and commenced CR in an inpatient setting within 30 days following discharge (Figure S1). The baseline characteristics of the analyzed groups are presented in Table 1. Patients who underwent outpatient CR were significantly younger and more frequently male compared to those participating in the inpatient program. The analyzed groups differed in several other variables. Using 1:2 propensity score matching, well-balanced groups with comparable covariates were formed (Table 1, Figure 1).

The mean follow-up duration was 332.8 (128.1) days (14,599.8 patient-years) for the entire study population and 335.3 (127.1) days for those who survived throughout the follow-up period. In total, 40 participants from the outpatient CR group and 288 participants from the inpatient CR group died during the observation period. Additionally, 146 outpatient CR participants and 535 inpatient CR participants experienced a myocardial infarction, while 18 and 88 patients, respectively, suffered a stroke. Furthermore, 1313 outpatient CR participants and 4118 inpatient CR participants required hospitalization for cardiovascular reasons at least once.

Table 2 presents variables independently associated with referral to and participation in outpatient CR. Younger age, male sex, a history of cancer, and hospitalization in a cardiology department were linked to a higher probability of participation in the outpatient program. Conversely, diabetes, heart failure, chronic kidney disease, chronic obstructive pulmonary disease, ST-elevation MI, and myocardial revascularization during the acute phase of MI were all independently associated with a higher probability of inpatient CR participation.

**Figure 2:**
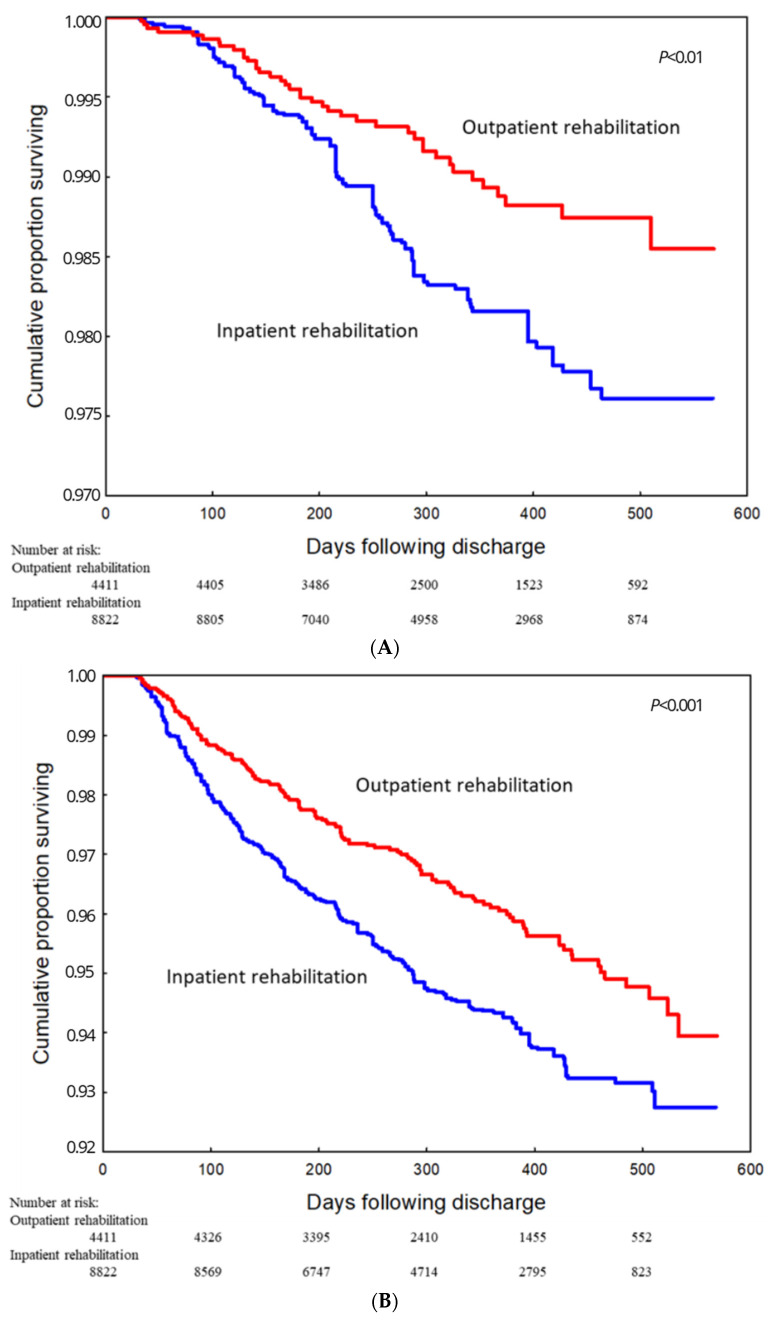

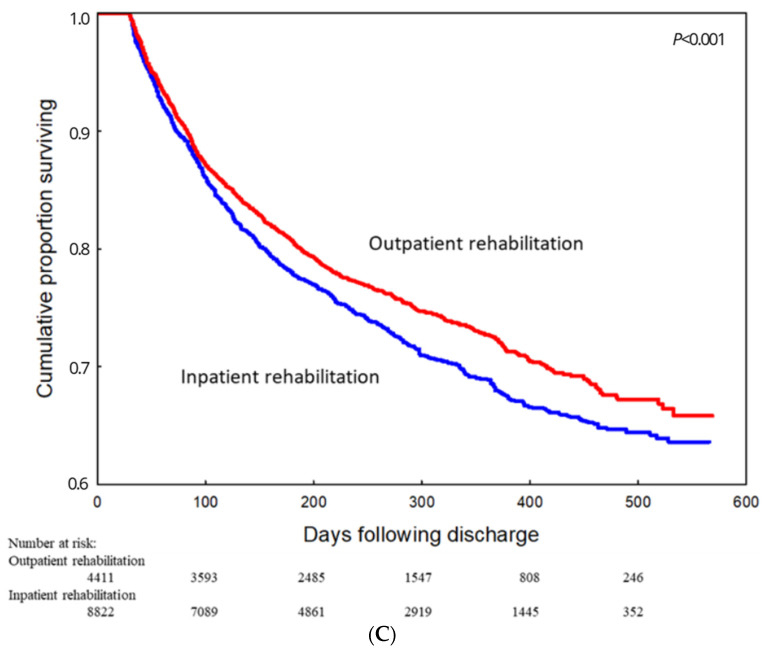
Kaplan–Meier curves displaying the estimated event-free survival probability of patients participating in the outpatient and inpatient cardiac rehabilitation programs following myocardial infarction. Matched groups. Panel (**A**): all-cause death. Panel (**B**): all-cause death, myocardial infarction, or stroke. (**C**): all-cause death or hospitalization for cardiovascular purposes.

One-year all-cause mortality was 1.1% among outpatient CR participants and 2.8% among inpatient CR participants (*p* < 0.001). When analyzing matched groups, all-cause mortality remained lower among outpatient CR participants (1.1% vs. 1.8%, *p* < 0.01; Figure 2A). The composite endpoint of all-cause death, MI, or stroke occurred in 3.9% and 7.0% of patients in the outpatient and inpatient CR groups, respectively (*p* < 0.001), in the unmatched analysis and in 3.9% vs. 5.7% (*p* < 0.001) when comparing matched groups (Figure 2B). The endpoint comprising all-cause death or hospitalization for cardiovascular reasons was observed less frequently in outpatient CR participants than in inpatient CR participants, both in unmatched groups (27.4% vs. 34.2%, *p* < 0.001) and in matched groups (27.4% vs. 31.4%, *p* < 0.001; Figure 2C). The proportional hazard (PH) assumption was met for the variable related to all-cause death or hospitalization for cardiovascular purposes. However, for the variables all-cause death and all-cause death, myocardial infarction, or stroke, residual results might suggest a slight time-varying effect.

The independent predictors of the study endpoints are presented in Table 3. Participation in outpatient CR was independently associated with all examined endpoints. Analysis of the unmatched groups revealed that participation in outpatient CR was associated with a reduced risk of death in both univariable analysis (hazard ratio [HR] [95% confidence interval (CI)] 0.37 [0.26–0.51]) and multivariable analysis (HR 0.53 [95% CI 0.38–0.74]). Outpatient CR participation was also associated with a lower risk of death, MI, or stroke (univariable: HR 0.57 [0.48–0.67]; multivariable: HR 0.72 [0.61–0.84]) and a lower risk of death or cardiovascular hospitalization (HR 0.78 [0.73–0.84] and HR 0.85 [0.80–0.91] for univariable and multivariable analyses, respectively). In the matched cohort, the HR (95% CI) was 0.57 (0.40–0.81) for all-cause death, 0.69 (0.57–0.82) for the composite endpoint (death, MI, or stroke), and 0.87 (0.81–0.94) for death or cardiovascular hospitalization. Table 4 presents the results of subgroup analyses.

As part of the sensitivity analysis, we repeated the analysis after excluding patients who experienced study endpoints within 90 days following discharge. Outpatient CR remained significantly associated with a reduced risk of death (HR 0.58 [0.40–0.83]), death, MI, or stroke (HR 0.69 [0.56–0.85]), as well as death or cardiovascular hospitalization (HR 0.85 [0.77–0.94]).

## 4. Discussion

Previous studies have shown that CR is associated with improved outcomes in patients with coronary artery disease, and clinical trials suggest that CR itself provides direct benefits [30]. According to the EUROASPIRE V study, approximately 34% of eligible coronary patients participate in CR programs after an acute event [31].

Only 13% of MI survivors started inpatient CR within 30 days following discharge. Additionally, 5% began outpatient CR within the same period. There are many barriers to participation in cardiac rehabilitation [32]. The lower proportion of outpatient CR participation among women compared to men, confirmed in our study, is consistent with some previous studies [33]. One of them showed that patient-level CR barriers of women included transportation, a high burden of family responsibilities, a lack of CR awareness, experience of exercise as tiring or painful, and comorbidities [34]. Older patients had significantly greater CR barriers to enrollment and participation in CR programs overall. This has been confirmed in our study as well as in other studies analyzing barriers to CR [32]. Norwegian studies [35] indicated a lack of referral routines and low availability as the main barrier provision. Only 18% of hospitals offered multidisciplinary cardiac rehabilitation in line with European recommendations [11], while 27% had ‘heart school’ classes without exercise training, and 8% had no provision for follow-up.

Although some patients started CR at a later period, phase 2 CR is known to be much more effective if started promptly following discharge from hospital [16,36]. Indeed, CR should commence as soon as possible after an acute event. In MI survivors, starting CR in the first 14 days following discharge is considered optimal, and the acceptable minimum is 15–30 days [16]. A total of 27.5% of rehabilitated patients took part in the outpatient program. Although, this proportion is low, it is higher than the proportion of patients receiving outpatient CR in Austria [37]. Participation in outpatient CR may be associated with reduced mortality and morbidity in MI survivors compared to inpatient CR. Several factors may explain this finding.

Despite efforts to account for confounding variables, unrecognized factors could influence the observed differences in event-free survival. Additionally, studies indicate that the number of attended CR sessions correlates with the effectiveness of rehabilitation [38]. Indeed, Suaya et al. found that patients who attended 25 or more CR sessions were 19% less likely to die over a five-year follow-up period compared to those who participated in 24 or fewer sessions [27]. Hammill et al. also demonstrated that mortality rates were consistently higher among patients who completed 12 or fewer sessions and lower among those who participated in 36 sessions [39]. These findings were confirmed by a meta-analysis, which showed that at least 36 sessions are required to reduce the need for coronary interventions [37]. The effectiveness of cardiac rehabilitation was also the subject of a British study [17]. The authors found that patients (N = 950) attending UK cardiac rehabilitation centers can only expect a 0.59 MET increase in their cardiorespiratory fitness over a typical 6–8-week program, which could be due to the lower number of supervised exercise sessions (6–16 compared to 36 in a systematic review of international trials) [40]. Furthermore, the beneficial effects on left ventricular remodeling are correlated with the duration of CR [41].

Although we were unable to assess the exact number of CR sessions attended, the average number of sessions in outpatient programs in Poland is higher than in inpatient programs. However, Poland has one of the lowest CR doses in Europe, primarily due to the widespread use of inpatient CR [15]. Studies have shown that when no supervision is provided following inpatient CR, the initial beneficial effects deteriorate within 12 months, with some parameters showing worse results at 12 months than at enrollment [42].

Our findings align with previous studies indicating better risk factor control among outpatient CR participants compared to those in inpatient CR [43]. The limited time available for physicians specializing in rehabilitation to implement lifestyle modifications or pharmacological interventions may partly explain these results [42].

Additionally, our findings are consistent with data from the Swiss AMIS Plus registry. Although the authors concluded that the risk of death was similar between inpatient and outpatient CR participants, they observed a trend toward a 39% lower mortality risk (*p* = 0.07) among outpatient CR participants, with an absolute difference closely resembling our findings [44].

To the best of our knowledge, this is the first study to directly compare the effects of outpatient and inpatient CR programs and the first to demonstrate a lower risk of cardiovascular events among outpatient CR participants compared to inpatient CR participants.

Although inpatient CR is considered particularly suitable for high-risk MI survivors, many low-risk patients are still referred to inpatient CR centers [10,37]. Importantly, we demonstrated that among patients referred to CR, a history of heart failure or stroke doubles the likelihood of participation in inpatient CR. The relatively weaker scientific evidence supporting the survival benefits of inpatient CR following MI has led some researchers to argue that the high prevalence of inpatient CR programs in certain countries is driven more by historical practice and reimbursement policies than by scientific evidence [14].

Recent evidence has further emphasized the prognostic relevance of in-hospital bleeding events in patients with acute coronary syndromes (ACSs). A recent study by Yudi et al. [45] demonstrated that bleeding complications during hospitalization are independently associated with increased mortality and adverse cardiovascular outcomes during follow-up. These events not only reflect a higher risk profile but may also contribute to delays or limitations in initiating secondary prevention strategies, including CR. Bleeding events may lead to reduced mobility, prolonged hospitalization, the interruption of antithrombotic therapy, and psychological distress, all of which can impair a patient’s ability or willingness to engage in structured CR programs. Moreover, clinicians may be more cautious in referring or enrolling patients with recent bleeding episodes into exercise-based interventions due to concerns about safety, despite the known long-term benefits.

While our multivariable analysis accounted for several clinical variables, we cannot exclude the possibility of residual confounding factors due to unmeasured factors, such as socioeconomic status, medication adherence, or unrecorded comorbidities. Additionally, although we used robust statistical techniques, selection bias may have influenced the observed associations, especially given the observational nature of the study. Obtained evidence of slight non-proportionality based on Schoenfeld residuals may reflect changing risk profiles over time. The potential for residual time-dependent confounding factors cannot be fully excluded and should be further explored in future studies.

### 4.1. Strengths and Limitations

This study has several key strengths. It included a nationwide, unselected patient cohort, making it representative of real-world clinical practice. The large sample size enabled robust multivariate analyses, while the registry encompassed a broad range of risk factors and covariates. Most of these criteria, as described above, have been met. Additionally, propensity score matching was performed to minimize bias. However, this study has several limitations. First, the design of the present study precludes any claims on cause-and-effect relations. Indeed, we could only confirm statistical associations between the analyzed variables, rather than a causal relationship. Second, this analysis did not include information on the dose and duration of exercise programs and cardiorespiratory fitness (Frequency, Intensity, Time, and Type—FITT), which are important for evaluating the quality and effectiveness of cardiac rehabilitation programs. Variations in these parameters across inpatient and outpatient settings could potentially impact outcomes and may account for some of the observed differences. Third, data on patients’ socioeconomic status (SES) were unavailable. SES is known to influence both access to healthcare services and long-term prognosis following myocardial infarction. Differences in income, education level, or social support might have contributed to selection bias or differential adherence to rehabilitation programs and thus may have influenced the comparative outcomes between inpatient and outpatient CR. Fourth, we were unable to account for other clinically relevant variables such as the use of cardioprotective medications (e.g., beta-blockers, ACE inhibitors, and statins), Killip classification, troponin levels, echocardiographic parameters (e.g., left ventricular ejection fraction), patients’ baseline lifestyle behaviors (e.g., smoking status, dietary habits, and physical activity), and the occurrence of adverse events during follow-up. The inclusion of such data would have allowed for more granular adjustment of confounders and may have enhanced the robustness and interpretability of our findings. Finally, this study’s conclusions depend on the reliability of the public databases used.

### 4.2. Clinical Implications and Future Research

The findings of this study highlight the potential benefits of outpatient cardiac rehabilitation following myocardial infarction and support its broader implementation as an effective component of secondary prevention. Given the association between outpatient CR and better clinical outcomes observed in this analysis, clinicians and healthcare systems should consider strategies to increase participation in outpatient programs, particularly for patients who are medically stable and are able to attend ambulatory sessions.

However, due to the observational nature of the study and the lack of detailed data on program characteristics and patient-level factors, caution is warranted in drawing causal conclusions. Future research should aim to address these gaps by incorporating comprehensive data on exercise prescription and patient response, including objective measures of cardiorespiratory fitness and detailed documentation of the FITT components. Additionally, the impact of socioeconomic factors, medication adherence, echocardiographic parameters, and patient lifestyle behaviors should be systematically evaluated to more accurately determine the drivers of improved outcomes.

Randomized controlled trials (RCTs) or well-designed prospective cohort studies comparing inpatient and outpatient CR, with standardized outcome measures and longer follow-up, are essential to validate our findings and better understand which patient subgroups benefit most from each type of program. Incorporating patient-reported outcomes and quality-of-life measures will also be critical in future investigations to assess the broader impact of CR models on functional recovery and well-being.

Ultimately, a more personalized approach to post-MI rehabilitation, guided by patient characteristics, preferences, and risk profiles, may optimize outcomes and resource utilization across diverse healthcare settings.

## 5. Conclusions

Patients who undergo outpatient CR after MI often show better outcomes than those in inpatient programs, although a randomized trial comparing outpatient and inpatient CR is warranted to further evaluate these findings.

## Figures and Tables

**Table 1:** Baseline characteristics of the analyzed groups.

	Patients Participating in Inpatient Rehabilitation	*p*	Patients Participating in Outpatient Rehabilitation	*p*	Matched Cohort of Patients Participating in Inpatient Rehabilitation
	N = 11,626		N = 4411		N = 8822
Age, years	64.8 ± 10.5	<0.001	61.5 ± 10.4	0.74	61.6 ± 11.0
Sex					
Males, n (%)	8077 (69.5)	<0.001	3324 (75.4)	0.13	6753 (76.5)
Females, n (%)	3549 (30.5)	<0.001	1087 (24.6)	0.13	2069 (23.5)
The patients’ history					
Hypertension, n (%)	7908 (68.0)	<0.001	2710 (61.4)	0.71	5449 (61.8)
Diabetes, n (%)	3068 (26.4)	<0.001	912 (20.7)	0.08	1940 (22.0)
Atrial fibrillation, n (%)	860 (7.4)	<0.001	231 (5.2)	0.85	469 (5.3)
Previous stroke, n (%)	225 (1.9)	<0.001	37 (0.8)	0.31	60 (0.7)
Previous myocardial infarction, n (%)	557 (4.8)	0.29	194 (4.4)	0.48	412 (4.7)
Previous PCI, n (%)	1010 (8.7)	0.01	328 (7.4)	0.74	670 (7.6)
Previous CABG, n (%)	92 (0.8)	0.56	31 (0.7)	0.82	59 (0.7)
Heart failure, n (%)	1491 (12.8)	<0.001	253 (5.7)	0.06	580 (6.6)
Chronic kidney disease, n (%)	500 (4.3)	<0.001	109 (2.5)	0.23	250 (2.8)
Previous dialysis, n (%)	35 (0.3)	0.02	4 (0.1)	0.84	9 (0.1)
Cancer in the history, n (%)	2454 (21.1)	0.57	950 (21.5)	0.86	1888 (21.4)
Chronic obstructive pulmonary disease, n (%)	1106 (9.5)	<0.001	254 (5.8)	0.94	511 (5.8)
Index hospitalization					
ST-elevation myocardial infarction presentation, n (%)	5133 (44.2)	<0.01	2051 (46.5)	0.07	4249 (48.2)
Coroangiography, n (%)	11,268 (96.9)	0.33	4263 (96.6)	0.07	8469 (96.6)
PCI, n (%)	10,201 (87.7)	<0.001	4101 (93.0)	0.28	8246 (93.5)
CABG, n (%)	1100 (9.5)	<0.001	23 (0.5)	0.80	49 (0.6)
Department					
Cardiology, n (%)	10,779 (92.7)	<0.001	4280 (97.0)	0.24	8591 (97.4)
Internal diseases, n (%)	617 (5.3)	<0.001	85 (1.9)	0.62	159 (1.8)
Other, n (%)	230 (2.0)	<0.001	46 (1.0)	0.19	72 (0.8)
Hospital					
Teaching, n (%)	2334 (20.1)	<0.001	1235 (28.0)	0.31	2396 (27.2)
District, n (%)	3815 (32.8)	<0.001	1865 (42.3)	0.27	3818 (43.3)
Municipal, n (%)	1876 (16.1)	<0.01	633 (14.4)	0.78	1285 (14.5)
Other, n (%)	3601 (31.0)	<0.001	678 (15.4)	0.61	1326 (15.0)

Values are presented as the mean ± standard deviation or n (%). PCI, percutaneous coronary intervention; CABG, coronary artery bypass grafting.

**Table 2:** Factors independently related to participation in the outpatient cardiac rehabilitation program following myocardial infarction (the whole cohort; n = 16,037).

Variable *	Odds Ratio (95% Confidence Intervals)
Age per 10 years	0.79 (0.77–0.83)
Male sex	1.32 (1.21–1.43)
Diabetes	0.88 (0.80–0.97)
Previous stroke	0.53 (0.37–0.76)
Heart failure	0.50 (0.43–0.58)
Chronic kidney disease	0.79 (0.63–1.00)
Cancer in the history	1.16 (1.05–1.27)
Chronic obstructive pulmonary disease	0.67 (0.58–0.79)
ST-elevation myocardial infarction presentation	0.84 (0.78–0.91)
PCI during the index hospitalization	0.63 (0.53–0.74)
CABG during the index hospitalization	0.04 (0.02–0.06)
Department	
Cardiology	1.63 (1.15–2.29)
Internal diseases	0.62 (0.41–0.93)
Other	1.00
Hospital	
Teaching	3.47 (3.10–3.88)
District	2.50 (2.25–2.76)
Municipal	1.76 (1.55–1.99)
Other	1.00

* the following variables were included in the statistical model: age, sex, hypertension, diabetes, atrial fibrillation, heart failure, stroke, MI, chronic kidney disease, dialysis, chronic obstructive pulmonary disease, a history of cancer, ST-elevation MI at presentation, coroangiography, PCI, CABG, hospital, and department.

**Table 3:** Independent predictors of all-cause death; all-cause death, myocardial infarction, or stroke; and all-cause death or hospitalization for cardiovascular reasons (the whole cohort; n = 16,037).

Variable	Hazard Ratio (95% Confidence Intervals)		
	All-Cause Death	All-Cause Death, Myocardial Infarction, or Stroke	All-Cause Death or Hospitalization for Cardiovascular Reasons
Age per 10 years	1.63 (1.44–1.83)	1.30 (1.22–1.39)	1.17 (1.14–1.20)
Sex, men—1, women—0	-	-	1.28 (1.21–1.36)
Hypertension	-	1.21 (1.03–1.43)	-
Diabetes	1.65 (1.32–2.07)	1.45 (1.27–1.65)	1.08 (1.01–1.14)
Atrial fibrillation	-	-	1.12 (1.01–1.24)
Previous stroke	1.85 (1.13–3.02)	-	-
Previous myocardial infarction	1.93 (1.40–2.64)	1.36 (1.04–1.76)	-
Previous PCI	-	-	1.19 (1.09–1.30)
Heart failure	2.28 (1.77–2.95)	1.67 (1.43–1.95)	1.18 (1.08–1.28)
Chronic kidney disease	1.57 (1.12–2.20)	-	-
Previous dialysis	-	3.17 (1.78–5.61)	1.69 (1.11–1.57)
ST-elevation myocardial infarction presentation	-	0.85 (0.74–0.97)	-
CABG during the index hospitalization	-	0.74 (0.57–0.97)	-
Hospitalization in a department of internal diseases	-	-	1.15 (1.02–1.29)
Hospitalization in a teaching hospital	-	-	0.91 (0.85–0.97)
Hospitalization in a municipal hospital	-	-	1.09 (1.02–1.17)
Outpatient cardiac rehabilitation	0.53 (0.38–0.74)	0.72 (0.61–0.84)	0.85 (0.80–0.91)

**Table 4:** Subgroup analysis of the relation between participation in the outpatient cardiac rehabilitation program and all-cause death and all-cause death, myocardial infarction, or stroke.

Variable	All-Cause Death		All-Cause Death, Myocardial Infarction, or Stroke	
	Hazard Ratio (95% Confidence Intervals)	*p* Value for Interaction	Hazard Ratio (95% Confidence Intervals)	*p* Value for Interaction
Age				
≤64 years	0.44 (0.24–0.82)	0.45	0.69 (0.54–0.89)	0.85
>64 years	0.57 (0.39–0.86)	0.45	0.74 (0.60–0.91)	0.85
Sex				
Males	0.44 (0.29–0.66)	0.18	0.71 (0.59–0.86)	0.96
Females	0.63 (0.45–1.14)	0.18	0.70 (0.51–0.72)	0.96
Hypertension No hypertension	0.61 (0.43–0.87) 0.14 (0.04–0.45)	0.02	0.72 (0.60–0.87) 0.70 (0.51–0.97)	0.86
Diabetes No diabetes	0.63 (0.39–1.02) 0.41 (0.26–0.65)	0.15	0.77 (0.59–1.01) 0.69 (0.57–0.84)	0.43
Atrial fibrillation No atrial fibrillation	0.15 (0.04–0.63) 0.56 (0.40–0.80)	0.13	0.30 (0.15–0.60) 0.77 (0.65–0.91)	0.02
Previous stroke No previous stroke	1.19 (0.25–5.58) 0.48 (0.34–0.67)	0.23	1.49 (0.54–4.09) 0.71 (0.60–0.84)	0.22
Previous myocardial infarction No previous myocardial infarction	0.69 (0.30–1.57) 0.46 (0.32–0.67)	0.29	0.77 (0.47–1.27) 0.71 (0.60–0.84)	0.84
Previous PCI No previous PCI	0.64 (0.32–1.31) 0.50 (0.34–0.73)	0.44	0.75 (0.51–1.12) 0.71 (0.60–0.85)	0.70
Heart failure No heart failure	0.54 (0.27–1.07) 0.52 (0.36–0.77)	0.74	0.85 (0.59–1.24) 0.69 (0.58–0.83)	0.23
Chronic kidney disease No chronic kidney disease	0.14 (0.02–1.02) 0.57 (0.41–0.81)	0.23	0.27 (0.10–0.74) 0.75 (0.63–0.88)	0.10
Cancer in the history No cancer in the history	0.37 (0.19–0.71) 0.60 (0.41–0.89)	0.33	0.67 (0.49–0.92) 0.73 (0.60–0.87)	0.59
Chronic obstructive pulmonary disease No chronic obstructive pulmonary disease	0.53 (0.19–1.48) 0.53 (0.37–0.76)	0.95	0.48 (0.24–0.96) 0.73 (0.62–0.87)	0.16
Index hospitalization				
ST-elevation myocardial infarction Non-ST-elevation myocardial infarction	0.44 (0.24–0.80) 0.58 (0.39–0.86)	0.44	0.72 (0.55–0.93) 0.72 (0.59–0.88)	0.90
PCI No PCI	0.55 (0.39–0.78) 0.28 (0.07–1.17)	0.42	0.75 (0.64–0.89) 0.40 (0.22–0.72)	0.15
Hospitalization in a department of cardiology Hospitalization in other departments	0.57 (0.41–0.80) 0.01 (0.00–430.1)	0.48	0.73 (0.62–0.85) 0.51 (0.20–1.28)	0.37
Hospitalization in a teaching hospital Hospitalization in other hospitals	0.49 (0.25–0.95) 0.54 (0.37–0.80)	0.90	0.71 (0.52–0.99) 0.72 (0.60–0.86)	0.71
Hospitalization in a district hospital Hospitalization in other hospitals	0.68 (0.42–1.10) 0.43 (0.27–0.69)	0.13	0.74 (0.58–0.95) 0.69 (0.56–0.85)	0.48
Hospitalization in a municipal hospital Hospitalization in other hospitals	0.57 (0.22–1.47) 0.52 (0.36–0.75)	0.85	0.70 (0.45–1.08) 0.71 (0.60–0.85)	0.92

## Data Availability

Data are available upon request from the corresponding authors.

## Supplementary Materials

The following supporting information can be downloaded at: https://www.mdpi.com/article/10.3390/jcm14093007/s1, Figure S1: Study groups.
