# Supplementary figures and images for: Comparison of Inpatient and Outpatient Cardiac Rehabilitation Following Myocardial Infarction
Source: J Clin Med. 2025 Apr 26;14(9):3007. doi: 10.3390/jcm14093007 (PMC12072414; doi:10.3390/jcm14093007)

Supplemental Figure S1. Study groups

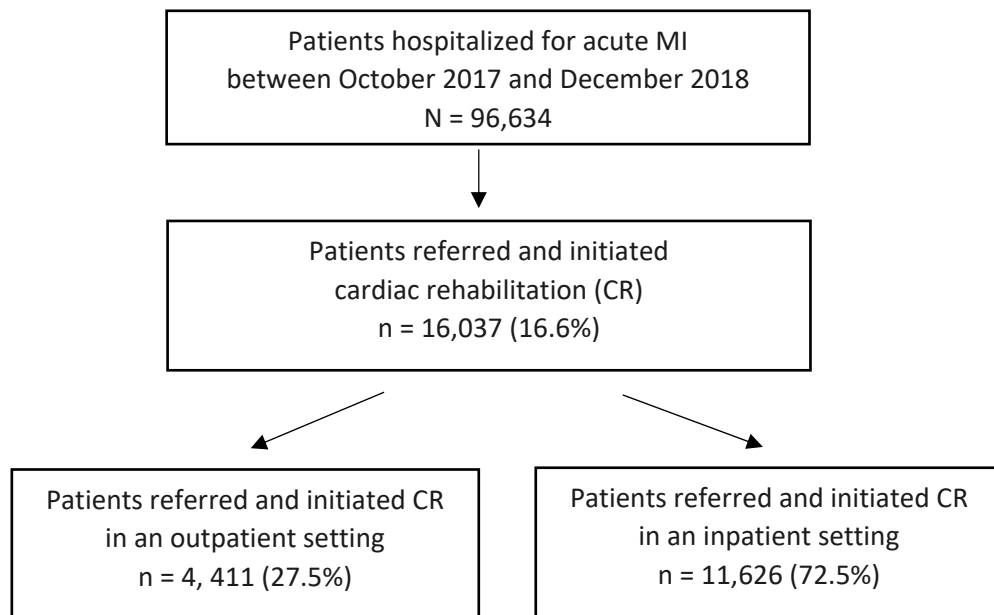

Supplement: Supplementary file 1 [file jcm-14-03007-s001.zip › jcm-3569164-supplementary.pdf]
